# Synthesis and Characterization of Chitosan-Based Nanodelivery Systems to Enhance the Anticancer Effect of Sorafenib Drug in Hepatocellular Carcinoma and Colorectal Adenocarcinoma Cells

**DOI:** 10.3390/nano11020497

**Published:** 2021-02-16

**Authors:** Umme Ruman, Kalaivani Buskaran, Giorgia Pastorin, Mas Jaffri Masarudin, Sharida Fakurazi, Mohd Zobir Hussein

**Affiliations:** 1 Materials Synthesis and Characterization Laboratory, Institute of Advanced Technology (ITMA), Universiti Putra Malaysia, UPM Serdang 43400, Selangor, Malaysia; chaity101@gmail.com; 2 Laboratory of Vaccine and Immunotherapeutic, Institute of Bioscience Universiti Putra Malaysia, UPM Serdang 43400, Selangor, Malaysia; vaneey_88@yahoo.com (K.B.); sharida@upm.edu.my (S.F.); 3 Faculty of Pharmacy, The National University of Singapore, 18 Science Drive 4, Kent Ridge, Singapore 117543, Singapore; phapg@nus.edu.sg; 4 Faculty of Biotechnology and Biomolecular Science, Universiti Putra Malaysia, Serdang 43400, Selangor, Malaysia; masjaffri@upm.edu.my; 5 Department of Human Anatomy, Faculty of Medicine & Health Sciences, Universiti Putra Malaysia, Serdang 43400, Selangor, Malaysia

**Keywords:** Sorafenib, chitosan-nanoparticles, folic acid, drug-delivery, therapeutic, HepG2, HT29, HDFa, cancer, cell lines

## Abstract

The formation of two nanodelivery systems, Sorafenib (SF)-loaded chitosan (SF-CS) and their folate-coated (SF-CS-FA) nanoparticles (NPs), were developed to enhance SF drug delivery on human Hepatocellular Carcinoma (HepG2) and Colorectal Adenocarcinoma (HT29) cell lines. The ionic gelation method was adopted to synthesize the NPs. The characterizations were performed by DLS, FESEM, TEM, XRD, TGA, FTIR, and UV-visible spectroscopy. It was found that 83.7 ± 2.4% and 87.9 ± 1.1% of encapsulation efficiency; 18.2 ± 1.3% and 19.9 ± 1.4% of loading content; 76.3 ± 13.7 nm and 81.6 ± 12.9 nm of hydrodynamic size; 60–80 nm and 70–100 nm of TEM; and FESEM sizes of near-spherical shape were observed, respectively, for SF-CS and SF-CS-FA nanoparticles. The SF showed excellent release from the nanoparticles under pH 4.8 PBS solution, indicating a good delivery system for tumor cells. The cytotoxicity study revealed their better anticancer action towards HepG2 and HT29 cell lines compared to the free sorafenib. Moreover, both NPs systems showed negligible toxicity to normal Human Dermal Fibroblast adult cells (HDFa). This is towards an enhanced anticancer drug delivery system with sustained-release properties for better cancer management.

## 1. Introduction

Nanodelivery platforms have been significantly manufactured using macromolecular chemistry through the manipulation of nanomaterials, especially, by the host-guest approach for the delivery of an anti-cancer drug. The development of nanodelivery systems loaded with therapeutic agents has opened better therapeutic efficacy for various cancer therapies. Polymeric materials-based nanocarriers have recently been developed for effective drug loading, controlled/sustained drug release, and sufficient drug accumulation in the site-specific disease areas. The current conventional cancer treatments such as extensive chemotherapy, radiotherapy, surgery, and immunotherapy are usually associated with high toxicity, drug losses, damage to healthy organs or cells, non-specific distribution, and tremendous side-effects, thus leading to a low rate of patient survival profile. The conventional chemotherapy for liver and colon cancer has many side-effects such as the unwanted distribution of drugs, less accumulation of drugs to the specific region of cancer, multiple drug resistance (MDR), high clearance rate, drug loss, etc. before it reaches the cancer cells. The therapeutic nanodelivery systems have come up with multiple positive impacts over conventional therapies for cancer. Due to the high-level efficiency of drug-loading, drug-encapsulation, effective intracellular uptake of the drug, controlled/sustained drug release, and drug accumulation within the cancer cells, the necessity for therapeutic delivery systems is high in clinical stages. Therefore, manufacturing more efficient and effective nanocarrier drug delivery systems to carry the drug to the specific affected areas or cancer cells with ample amounts of drug and excellent therapeutic duration period is necessary to overcome the chemotherapeutic side-effects in the human body [1].

Liver cancer is one of the most deadliest cancers, causing a great number of deaths worldwide. Hepatocellular carcinoma (HCC) accounts for most liver cancers [2]. On the other hand, colon cancer is the third-most diagnosed cancer and the fourth cause of cancer death worldwide [3]. Therefore, novel preventive strategies are needed to put into practice to treat liver and colorectal cancer [4]. In 2018, liver cancer caused 781,631 (8.2%) deaths and colon cancer caused 551,269 (5.8%) deaths all around the world [5]. Nanoscale drug delivery systems (NDDSs) will overcome the toxicity, reduce drug dose, increase drug functionality, and lessen other side-effects or barriers. NDDSs can encapsulate, entrap, bind, absorb, adsorb, attach, and carry various compounds such as drugs, genes, theranostic agents, probes, proteins, and targeting moieties, depending on the interaction between the host and guest, which, in this case, is the tumor cells, and then release it at the tumor site so that less healthy cells will be affected by the drugs [6]. Thereby, nanodelivery systems can be engaged to help the drug or therapeutic molecules to reach the specific disease site and protect the drugs from biological barriers in the body [7,8].

Chitosan polymer nanoparticles (CS-NPs) is a promising nanodelivery system. CS is a cationic biopolymer that can be prepared from the deacetylation of chitin. It is a linear polysaccharide chain of 1-4,-linked-D-Glucosamine and N-acetyl-D-glucosamine. CS-NPs were found to be a safe and effective drug transport agent to cancer cells [9]. Studies have shown that CS-NPs have better bioactivity, solubility, biodegradability, biocompatibility, and less toxicity [10,11].

Sorafenib (SF) is a chemotherapeutic agent used for liver and colon cancer therapy. SF was the first drug approved in 2007 by the US Food & Drug Administration (FDA) to treat liver cancer patients. The national comprehensive cancer network and the American Association has recommended SF as a first-line drug for hepatocellular carcinoma (HCC) [12,13,14]. SF leads to the inhibition of HCC tumor cell proliferation as well as invasion by inhibiting the cell proliferation, angiogenesis, and threonine kinase activities in tumor areas in multiple oncogenic signaling pathways. SF targets the multiple kinase protein B-Raf and C-Raf, Vascular Endothelial Growth Factor Receptor 2 (VEGFR2), and Platelet-derived Growth Factor Receptor (PDGFR), which are responsible for HCC [15,16]. SF enhances the production of O_2_ˉ, NO, and H_2_O_2_ in human HepG2 cells. Reactive oxygen species (ROS) causes oxidative damages to cancer cell’s DNA, proteins, lipids, and eventually leads to cell death [17]. It is a hydrophobic drug that is poorly soluble (25 mg/mL) in deionized water [18,19,20]. The recommended dose of SF for HCC therapy is 400 mg. However, low bioavailability and severe side-effects have been reported at this dosage, limiting the clinical application of SF [21]. Due to low bioavailability, the long-term chemotherapy of this drug with high dosage may restrict therapeutic activities and cause greater suffering to the patients. Therefore, a study to develop nanodelivery systems for SF is urgent to overcome the resistance of SF on cells and severe side-effects of the SF drug on normal cells/tissues, thereby improving anticancer therapeutic efficacy [22]. SF responds to colon cancer as well, which was found in previous work on HT29 and SW480 cell lines. The studies found that cellular proliferation and cancer cell growth is inhibited by sorafenib-treated HT29 cells [23]. SF embedded in liposome nanocarrier has shown good anticancer outcomes on colon cancer cells [24]. On the other hand, SF-loaded PEG-PLGA nanoparticles showed high uptake of SF by colorectal cancer cells and increased anticancer action compared to free drugs [25].

The expression of folate receptors (FRs) is 100–300 times higher in the epithelial liver and colon cancer/tumor cells compared to normal cells [26]. Therefore, folic acid (FA) can be attached to the surface layer of the nanoparticles as a ligand to deliver chemotherapeutic drugs targeting the cancer cells. The binding efficacy of FA to these FRs of liver/colon cancer cells is high. Therefore, folate can be used as an ideal ligand to perform active targeting in nanodrug delivery vehicles of liver/colon cancer cells [27,28,29]. Folate can enter cells through a receptor-mediated endocytosis pathway. The endocytosis mediated FRs is a good target in liver/colon tissues to absorb the FA conjugate nanocarrier effectively [30,31]. 

This study aims to optimize the formulation of SF-loaded, SF-CS, and folate-coated SF-CS-FA nanoparticles to enhance the drug loading, encapsulation, sustained release, and anticancer effects of sorafenib drug to liver/colon cancer cell lines by manipulating the amount of chitosan, sorafenib, TPP, and folic acid. The SF-CS and SF-CS-FA nanoparticles were synthesized via the ionic-gelation method based on the interaction or crosslinking between the negatively charged group of phosphate in sodium tripolyphosphate (TPP) and the positively charged group of the amine in chitosan. The nanoparticles can be customized by modifying the cross-linking parameters between chitosan chains and their interaction with TPP, as well as by optimizing the ratio of chitosan and sorafenib to enhance the therapeutic payload. The amount of folic acid was also customized to enhance the nanoparticles’ efficiency to drug load and encapsulation. The resulting nanoparticle’s effectiveness will be tested by various physico-chemicals characterizations, in vitro release, and anticancer activity by MTT assay on liver and colon cancer cell lines. 

## 2. Materials and Methods

### 2.1. Materials

Chitosan (low molecular weight, deacetylation 75–85%) and Sorafenib drug were purchased from Sigma Aldrich (Saint Louis, MO, USA). Sodium Tripolyphosphate (TPP) was purchased from Merck and acetic acid (99.8%) was purchased from Hamburg Industries, Inc. (Hamburg, Germany). Folic acid, Tween 80, dimethyl sulfoxide (CH_3_)_2_SO were obained from Sigma Aldrich (Hamburg Industries Inc., Hamburg, Germany). Deionized water (18.20 MΩ·cm^−1^) was used throughout the experiment. Primary Dermal Fibroblast: Normal, Human, Adult (HDFa) (ATCC^®^ PCS-201-012™), Hepatocellular carcinoma cell, (HepG2-ATCC^®^ HB-8065™), Colorectal adenocarcinoma (HT29-ATCC^®^ HTB-38™) were purchased from ATCC (AMERICAN TYPE CULTURE COLLECTION, PO BOX 1549, manassas, VA 20108, USA).

### 2.2. Synthesis of Sorafenib-Loaded Chitosan Nanoparticles

**Figure 1:**
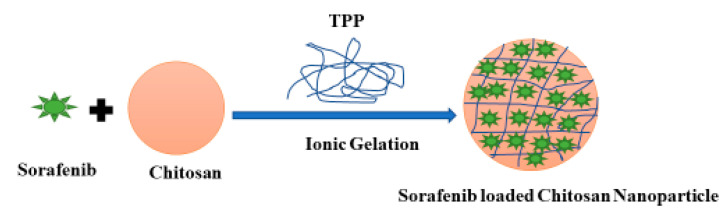
Schematic diagram of the synthesis steps of Sorafenib-loaded chitosan nanoparticles.

Sorafenib-loaded chitosan nanoparticles (SF-CS) were prepared via the ionic gelation technique. A solution of 1.0% (*v/v*) acetic acid to dissolve 5.0 mg/mL of chitosan powder and 0.3 g of DMSO to dissolve sorafenib drug were prepared and mixed with vigorous stirring. The pH of the solution was fixed at 5, by adding the NaOH solution (1 M). About 2% *v/v* of TWEEN-80 was added as a surfactant into the solution. About 7 mg/mL sodium tripolyphosphate (TPP) solution was prepared in dH_2_O water separately and pH was fixed to 2 using HCl (1 M). Using a burette, TPP solution was added dropwise into the chitosan-sorafenib solution while maintaining vigorous stirring. The resulting suspension was subsequently centrifuged at 4000 rpm and washed with deionized water and the sample was finally freeze-dried for further characterization and cytotoxicity studies. SF-CS NPs were used to administer the SF via the passive accumulation into the cancer cells. Figure 1 shows a schematic diagram of the synthesis steps of Sorafenib-loaded chitosan nanoparticles (SF-CS NPs).

### 2.3. Preparation of Folic Acid-Conjugated Chitosan Sorafenib Nanoparticles

**Figure 2:**
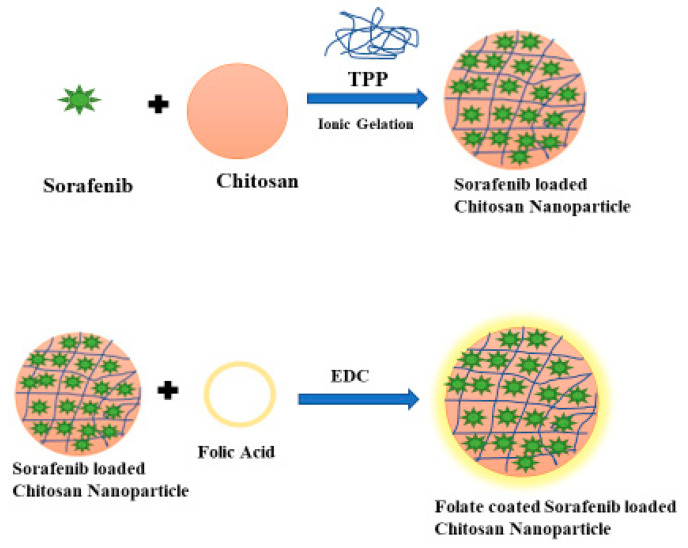
Schematic diagram of the synthesis steps of folic acid-conjugated chitosan Sorafenib nanoparticles.

Folic acid (FA) was conjugated with sorafenib chitosan nanoparticles, forming folate-conjugated, sorafenib-loaded chitosan nanoparticles (SF-CS-FA) for active targeting of cancer cells. SF-CS-FA nanoparticles were prepared by mixing 5.0 mg/mL of chitosan powder in a 1.0% (*v/v*) acetic acid solution. About 0.3 g of Sorafenib was dissolved in DMSO and added to the chitosan solution followed by controlling the pH to 5. Then, 2% *v/v* of TWEEN-80 was added into the chitosan-drug solution. About 7 mg/mL of the TPP solution was prepared in dH_2_O separately, at pH = 2 and was added dropwise into the chitosan solution with vigorous stirring. The NPs were formed by the ionic gelation method. To coat the NPs with FA, a solution of 0.8 g FA and 0.25 g of EDC (1-ethyl-3-(dimethyl aminopropyl) was prepared and stirred at room temperature until the EDC and folic acid were well-dissolved and mixed. The mixture was then added slowly into the chitosan-drug solution and stirred for 48 h at room temperature in the dark to let the folic acid conjugate onto chitosan molecules. Then the sample was centrifuged at 4000 rpm and washed with dH_2_O, and freeze-dried for further characterizations and cytotoxicity studies. Figure 2 shows a schematic diagram of the synthesis steps of folic acid-conjugated chitosan sorafenib nanoparticles (SF-CS-FA NPs).

### 2.4. Physico-Chemical Characterization

The particle size distribution, polydispersity index (PDI), Zeta potentials of SF-CS, and SF-CS-FA nanoparticles were measured by a Nanosizer, NanoS, Malvern Panalytical Ltd.; Malvern WR14 1XZ, United Kingdom UK.

The x-ray diffraction (Shimadzu XRD 6000, Kyoto 604-8511, Japan) was used to analyze the crystalline phase of the sorafenib drug, CS-NPs, SF-CS, SF-CS-FA nanoparticles in 2–40° range using CuK_α_ radiation (λ = 1.54060 Å) at 40 kV and 30 mA.

The morphological characteristics and size distribution were determined by a transmission electron microscope (TEM, Hitachi H-7100, Tokyo, Japan) at 100 kV accelerating voltage. The particle size distribution was analyzed by an image analysis software (UTHSCSA Image Tool V.6). A drop of dilute SF-CS, SF-CS-FA nanoparticles solutions were placed on carbon films, 300 mesh copper grids. The samples were air-dried before observation by TEM.

The thermal decomposition of the nanoparticles was determined by thermogravimetric/differential thermogravimetric analysis (TGA/DTG) using a Mettler-Toledo instrument (Greifensee, Switzerland) at 25–1000 °C with 10 °C min^−1^ heating rate.

A field emission scanning electron microscope (FESEM, NOVA NANOSEM 230, Nova™ NanoSEM 230 - FEI Company, CA, USA) was used to study the shape and morphology of SF-CS, SF-CS-FA nanoparticles. The liquid solutions of the nanoparticles were put on the top of a stub and dried in an oven and analyzed.

The composition of the prepared SF-CS, SF-CS-FA nanoparticles was analyzed by Energy Dispersive X-Ray spectroscopy in conjunction with FESEM (FESEM-EDX, NOVA NANOSEM 230, Nova™ NanoSEM 230 - FEI Company, CA, USA).EDX was used to determine the atomic and weight percentages of elements that exist in the sample such as oxygen, nitrogen, hydrogen, sulfur, and carbon compounds and the percentages were calculated using the EDX spectrum.

The FTIR analysis of SF, CS nanoparticles, SF-CS, SF-CS-FA nanoparticles was analyzed using a FTIR spectrometer (Perkin Elmer Spectrum RX FT-IR FTIR Spectrum RX, SPECTRUM 1000, PerkinElmer, Manasquan, NJ, USA). A small portion of the sample (5.0–8.0 mg) was put on a holder plate and subjected to light within the infrared spectrum. The instrument operated with a resolution of 4 cm^−1^. The FTIR spectra scans were analyzed between 500 and 4000 cm^−1^.

### 2.5. Encapsulation Efficiency (EE%) and Loading Content (LC%)

An ultraviolet-visible spectrophotometer (UV-Vis Spectrum) was used to determine the percentage of SF loading content (% LC) and encapsulation efficiency (% EE) using maximum wavelength of λ_max_ = 265 nm for SF drug. Briefly, 0.1 g of each nanoparticle was dissolved separately in 10.0 mL methanol with 0.5% (*v/v*) of hydrochloric acid (HCl). After 20 min of sonication, a crystal-clear solution was achieved prior to the UV-vis analysis. The nanoparticles were confirmed to be totally dissolved and released 100% of SF content. The encapsulation efficiency (% EE) and loading content (% LC) of SF-CS, SF-CS-FA nanoparticles were calculated using the following formula:

(1) $$\mathrm{EE}\;\left(\%\right)\;=\;\frac{\mathrm{Total}\;\mathrm{nanoparticle}\;\mathrm{with}\;\mathrm{drug}\;-\;\mathrm{Free}\;\mathrm{drug}}{\mathrm{Total}\;\mathrm{nanoparticles}\;\mathrm{with}\;\mathrm{drug}\;}\;\times \;100\%$$

(2) $$\mathrm{LC}\;\left(\%\right)\;=\frac{\mathrm{The}\;\mathrm{weight}\;\mathrm{of}\;\mathrm{sorafenib}\;\mathrm{in}\;\mathrm{nanoparticles}\;}{\mathrm{The}\;\mathrm{weight}\;\mathrm{of}\;\mathrm{nanoparticles}\;}\times \;100\%$$

### 2.6. In Vitro Drug Release Study

The release pattern of SF drug from the SF-CS, SF-CS-FA nanoparticles were evaluated for 5 days. In brief, 10 mg of nanoparticles were suspended in 10 mL PBS buffer solutions of pH = 7.4 and 4.8 and placed in an orbital shaker at 300 rpm at 37 °C. The samples were taken for analysis at specified time intervals; 1, 2, 3, 4, 5, 6, 7, 8, 12 h, and 2, 3, 5, 6 days incubation. Then the drug concentration was determined using UV-Vis spectroscopy. The drug release patterns of the SF-CS, SF-CS-FA nanoparticles were analyzed.

### 2.7. In Vitro Cell Viability Assay

The toxicity study of SF-CS and SF-CS-FA NPs was conducted by cell viability assay. Three types of cell lines; human hepatocellular carcinoma cells (HepG2), human colorectal adenocarcinoma cell lines (HT29), and the normal human dermal fibroblast cells (HDFa) were used for the cytotoxicity assay. All the cells were sourced from ATCC (AMERICAN TYPE CULTURE COLLECTION, PO BOX 1549, Manassas, VA 20108, USA). The HDFa dermal fibroblast was grown in fibroblast basal medium with fibroblast growth kit–serum-free (ATCC^®^ PCS-201-040), while, HepG2 cells were grown in Eagle’s minimum essential medium, and HT29 cells were grown in McCoy’s 5a modified medium with 10% supplement of fetal bovine albumin (Sigma-Aldrich, MO, USA) and 1% penicillin antibiotics containing 10,000 units/mL and 10,000 μg/ mL of streptomycin (Nacalai Tesque, Kyoto, Japan). All the cells were maintained and incubated at 37 °C in 5% humidified carbon dioxide (CO_2_) followed by harvesting using 0.25% of trypsin/1mM-EDTA (Nacalai Tesque, Kyoto, Japan). In an incubator, the cells were seeded in 96-well plates at 1.0 × 10^4^ cells/well for 24 h until 80% of confluence was attained for the treatment. Methylthiazol tetrazolium (MTT) assay was carried out to investigate cell cytotoxicity and viability. Cells were treated with chitosan (CS), Sorafenib (SF), chitosan-sorafenib-nanoparticles (SF-CS nanoparticles), and chitosan-sorafenib-folic acid-coated nanoparticles (SF-CS-FA nanoparticles).

The stock solutions were prepared by dissolving the samples in RPMI and 1:1 of dimethyl sulfoxide (0.1%). The solutions were then diluted to make various concentrations from 1.25 to 100 μg/mL in the same media. After 24 h, when the cells were attached to the respective wells, the prepared sample solution was further added to the wells until the final volume was obtained at around 100 μL. After 72 h of incubation, 10 μL of MTT solution (5 mg/mL in PBS) was added in each well and incubated for 3 h. After that, 100 μL of dimethyl sulfoxide was added per well at room temperature in the dark to dissolve the purple formazan salt. The intensity of the purple formazan solution was subsequently measured at a wavelength of 570 nm using a microplate reader (BioTek Instruments, Inc., Winooski, VT, USA, which reflected cell growth. The cytotoxicity assays were conducted in triplicates. Standard deviations were calculated and incorporated in respective bar graphs. Statistical analysis was performed by Analysis of Variance (ANOVA), followed by the Duncan’s Multiple Range Test, if a significant difference was observed using SPSS program Version 22.

## 3. Results and Discussion

### 3.1. Optimization of Particle Size, Poly Dispersity Index, Encapsulation and Loading Efficiency, and Zeta Potentials

Low molecular weight chitosan powder was chosen due to its excellent biodegradability and biocompatibility properties [10,11]. In this study, all the nanoparticles were formed through the ionic cross-linking between chitosan chains and TPP. The cross-linking reaction occured due to the ionic interaction with protonated primary amino groups of chitosan and phosphate groups of TPP. The drug became physically entrapped when the cross-linking occurred between chitosan and TPP. Tween 80, a non-ionic surfactant was used to alter the particle surface as well as improve the sphericity of the nanoparticle and make the surface smooth. EDC was used to conjugate FA onto the nanoparticle’s surface. The pH of the CS drug solution was fixed at pH = 5, the optimum pH for better chitosan-TPP nanoparticles formation [32].

The drug loading in the nanoparticles depends on factors such as the nature of the drug, the carrier polymer, and the surfactant [33]. The drug, SF loaded in the CS nanoparticles, was determined using the UV-Vis. SF loading percentage depends on the chitosan polymer concentration. The optimum ratio of chitosan powder, sorafenib drug, and TPP concentration was fixed before it is coated with folic acid. It seems that the percentage of chitosan polymer should be higher than the drug to increase the interactions between chitosan and SF, which may result in a higher concentration of drug encapsulation and loading. Increasing the TPP concentration will decrease the size; as a result, the drug may not be entrapped in chitosan chains. Therefore, optimum TPP concentration is needed to get drug entrapment.

Zeta analysis was carried out to investigate the nanoparticle’s changes in surface charges. A positive zeta potential indicates the presence of amine groups in CS. As surface charge affects various physio-chemical activities, positively charged surface impacts the better stability of NPs [34]. However, PDI values describe the stability of the particles; the lower the PDI, the better the uniformity of the particle. The stability of the NPs system is related to the PDI index value, which is not affected by the zeta potential charges, as nanoparticles with low PDI value have better stability, compared to high PDI with lower stability [35]. The nanoparticles reached optimum particle size and drug loading at 7 mg/mL of TPP, 0.5 g of CS, and 0.3 g of SF at pH 5.

**Figure 3:**
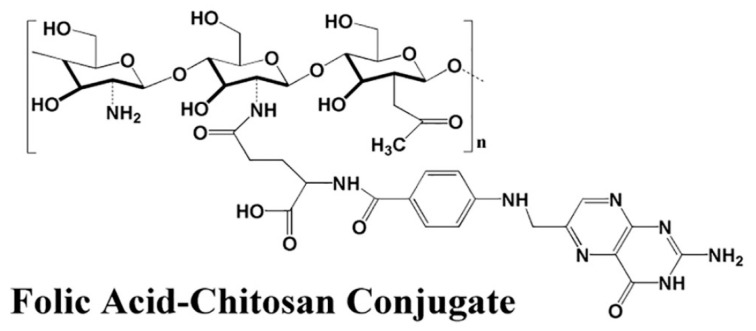
Formation of chitosan-folic acid conjugation through the amine group of chitosan and OH group of FA.

**Figure 4:**
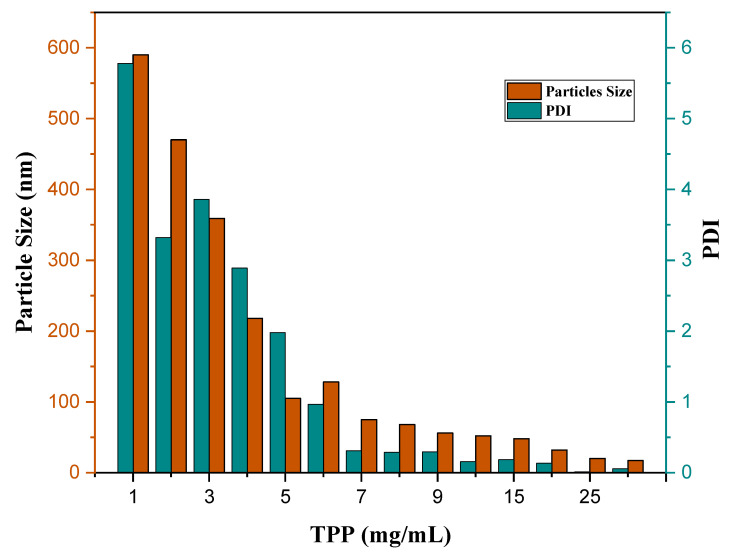
The effect of TPP (mg/mL) on particle size (nm) and PDI index of SF-CS NPs.

The folic acid amount was optimized by manipulating the amount of folic acid concentration. It was found that increasing the folic acid concentration also increases the coating efficiency, and optimum size and PDI. Table 1 represents the effect of size, PDI, % EE and % LC, and zeta potential on the chitosan:Sorafenib ratio. Table 2 shows the effect of the size, PDI, and zeta potential on the change of folic acid concentration. It was found that increasing folic acid concentration will increase the size. A lower concentration of folic acid shows no coating effect. The ratio of folic acid should be higher than the chitosan and drug. In this work, the best coating effect was observed when the amount of CS, SF, and FA was at 0.5, 0.3, and 0.8 g/L, respectively. The EDC amount used was fixed at 0.25 g in all the parameters, to open the carboxylic group in chitosan. The chitosan was conjugated with folic acid with the amine group of chitosan and OH group of FA. Figure 3 shows the conjugation of folic acid to chitosan polymers. Figure 4 has shown the effect of TPP on particle size (nm) and PDI index of SF-CS NPs which noted that the increasing TPP concentration will decrease the size and PDI. Table 3 has shown the percentages of LC and EE of SF-CS and SF-CS-FA NPs at the optimum amount of CS, SF, and FA.

### 3.2. Particle Size Distribution

Particle size is a crucial attribution to a nanocarrier. It often affects the cellular uptake of nanocarrier internalization to cells. This is the preliminary consideration for a nanocarrier’s therapeutic impact. Particle size distribution was analyzed by the DLS technique. PDI > 0.7 indicates the broad particle size distribution of the sample, which may not be suitable for analysis by the DLS technique. PDI ≤ 0.2 value of polymer-based nanoparticles such as chitosan is deemed suitable for therapeutic purposes. PDI ≤ 0.3 represented homogenous nanocarrier vesicles, which are considered as an effective nanocarrier for cell internalization [36,37].

**Figure 5:**
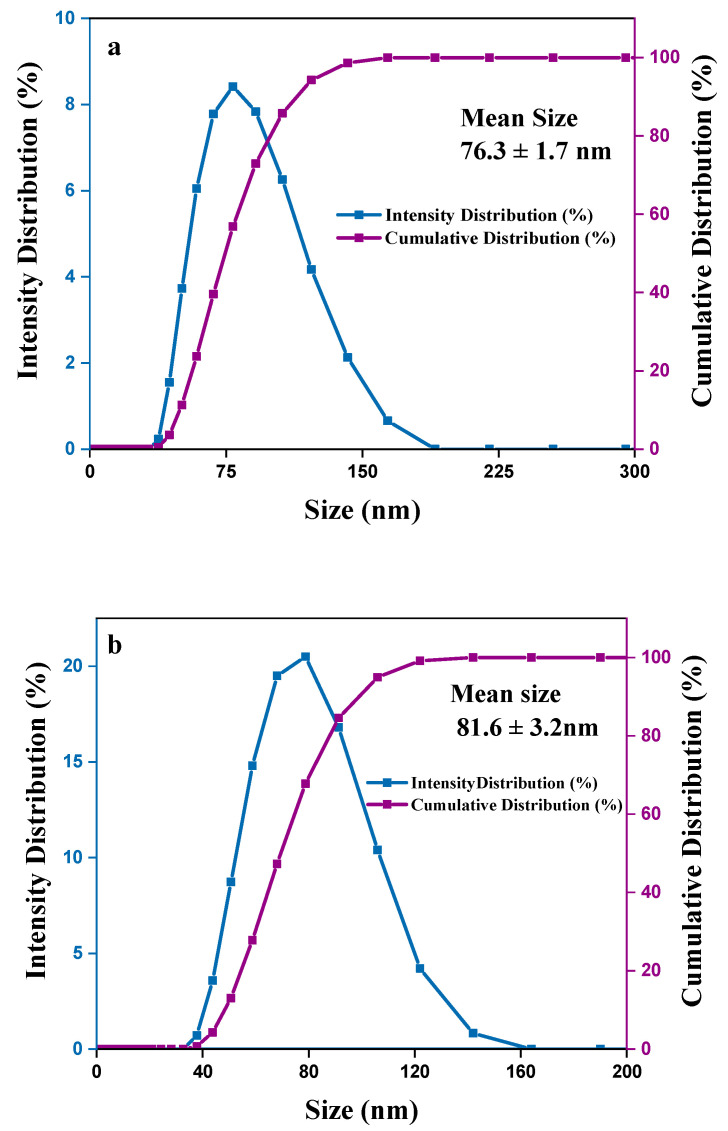
Particles size distribution by the intensity and cumulation of (**a**) sorafenib-loaded chitosan nanoparticles (SF-CS) and (**b**) folate-conjugated sorafenib-loaded chitosan nanoparticles (SF-CS-FA).

In the optimum condition according to Table 1 and Table 2, and Figure 4, the average mean particle size of SF-CS nanoparticles was found to be 76.3 ± 1.72 nm compared to 81.6 ± 7.9 nm for its FA-conjugated nanoparticles, with excellent dispersity index (PDI ~ 0.20) of the latter (Figure 5). The covalent conjugation of FA as a ligand to nanoparticles might be responsible for the larger particle size. In passive targeting, by enhanced permeability and retention effect (EPR), the particle size of 100 nm–2 µm will allow specific uptake and accumulation in tumor tissues, due to the leaky vasculature and defective lymphatic drainage of cancer cells [38]. For active-targeting, nanoparticles of around 100–200 nm could play a vital role in maintaining particle stability and influence cellular internalization in the cancer cells [39,40]. In the DLS method, the hydrodynamic size of particles was measured after the drug loading and the resulting nanoparticles were found to be larger, due to the interaction between Sorafenib and chitosan polymer chains as well as folate conjugation. Figure 5 represents the hydrodynamic particle size of SF-CS and SF-CS-FA NPs, synthesized at the optimum parameters, where the chitosan-to-sorafenib ratio of 5:3, folic acid 0.8 g with pH 5, TPP 7 mg/mL, and PDI ≤ 0.3 was used. Figure 5a,b show the intensity and cumulative size distribution of SF-CS and SF-CS-FA nanoparticles, respectively.

### 3.3. X-ray Diffraction

**Figure 6:**
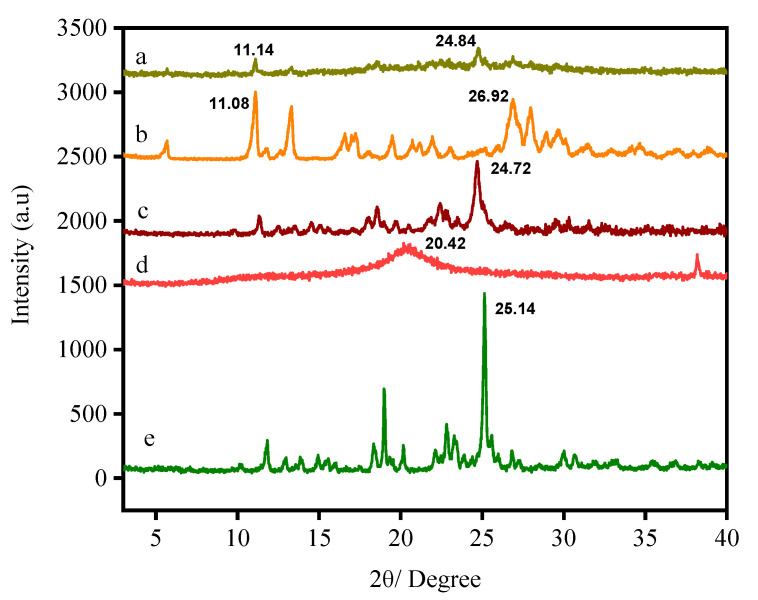
XRD patterns of folic acid-coated chitosan-loaded sorafenib nanoparticles (**a**), folic acid (**b**), Sorafenib-loaded chitosan nanoparticles (**c**), chitosan nanoparticles (**d**), and the drug Sorafenib (**e**).

The X-ray diffraction patterns of SF, FA, CS, CS NPs, SF-CS NPs, and SF-CS-FA NPs are shown in Figure 6. Figure 6e represents the XRD pattern of free SF, which shows a sharp reflection at 2θ = 25.1° and other several reflections, suggesting their highly crystalline nature. In contrast, chitosan exhibits a broad reflection and is amorphous. XRD patterns of chitosan (CS) shows a sharp reflection at 2θ = 20.4° (Figure 6d). Figure 6c shows a slight decrease in the crystalline nature when Sorafenib-loaded chitosan nanoparticles were formed. The increase in the crystallinity of Sorafenib-loaded chitosan nanoparticles was observed compared to chitosan alone due to a drug-loaded episode. For the SF-CS nanoparticles, the crystalline reflections of SF were observed to be embedded in the amorphous chitosan phase. A few sharp reflections at 2θ of 12.3, 18.1, 24.7, and 29.9° matched with SF with slight left shifting compared to the reflection patterns of free SF, thus confirming the loading of SF onto the chitosan polymer matrix. A few reflections (Figure 6b) can be observed, indicating the semi-crystalline nature of the FA. Figure 6a shows a slight decrease in crystalline nature when Sorafenib was loaded into the chitosan nanoparticles and coated with FA. Figure 6a shows several sharp reflections at 2θ of 5.9, 11.12, 14.2, 16.1, 23.8, 27.1, and 28.5°, matched with the reflections pattern of free FA with slight shifting, thus indicating that the coating of FA in the chitosan drug matrix was accomplished.

### 3.4. Surface Properties Using Field Emission Scanning Electron Micrographs and Qualitative Elemental Analysis Using Energy Dispersive X-ray

**Figure 7:**
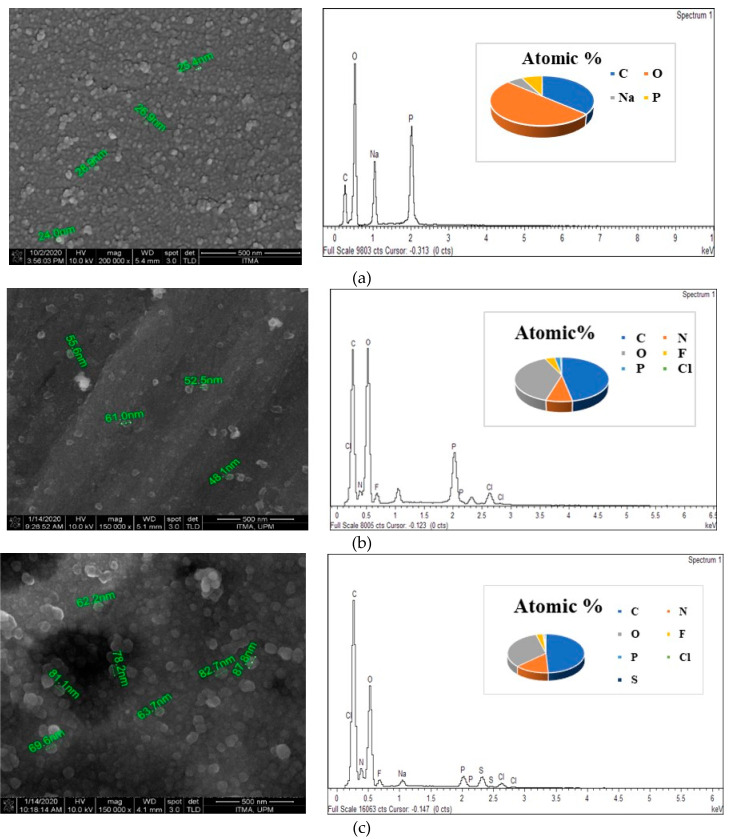
FESEM images and EDX spectrum of (**a**) CS-NPs, (**b**) SF-CS nanoparticles, (**c**) SF-CS-FA nanoparticles.

The surface morphology of the synthesized nanoparticles was observed by the field emission scanning electron micrographs (FESEM) technique. Energy Dispersive X-Ray Analysis (EDX) in conjunction with FESEM was conducted to investigate the compositional analysis of SF-CS and SF-CS-FA nanoparticles. Figure 7 shows the FESEM image and EDX compositional elements of SF-CS and SF-CS-FA nanoparticles. FESEM images show the near-spherical shape of the nanoparticles with a uniform structure. Figure 7a shows the pure chitosan nanoparticles distribution with a size range of 20–30 nm. Figure 7b shows SF-CS NPs with a size around 40–70 nm, which is due to the drug encapsulation. Figure 7c is folate-coated, SF-CS-FA nanoparticles with a relatively bigger size of 60–120 nm. This drug loading as well as FA coating is responsible for the bigger size. All the synthesized nanoparticles show spherical shape with narrow size distribution and relatively smooth surface.

The EDX elemental composition of weight and atomic percentages of all the samples are shown in Table 4. Different areas were focused on during the EDX measurement to get their corresponding elemental contents. The presence of C, N, P, O, F, and Cl was found in all the samples. Atomic percentages of carbon and oxygen exhibited the highest amount in all the samples. Weight percentages of C, N, O, F, P, Cl, and S were present in the synthesized SF-CS and SF-CS-FA nanoparticles. Sulfur was present due to the existence of folic acid in the coating layers. In pure CS-NPs, sodium was present in a large amount due to the existence of sodium-tripolyphosphate during the crosslinking process.

### 3.5. High-Resolution Transmission Electron Micrograph

**Figure 8:**
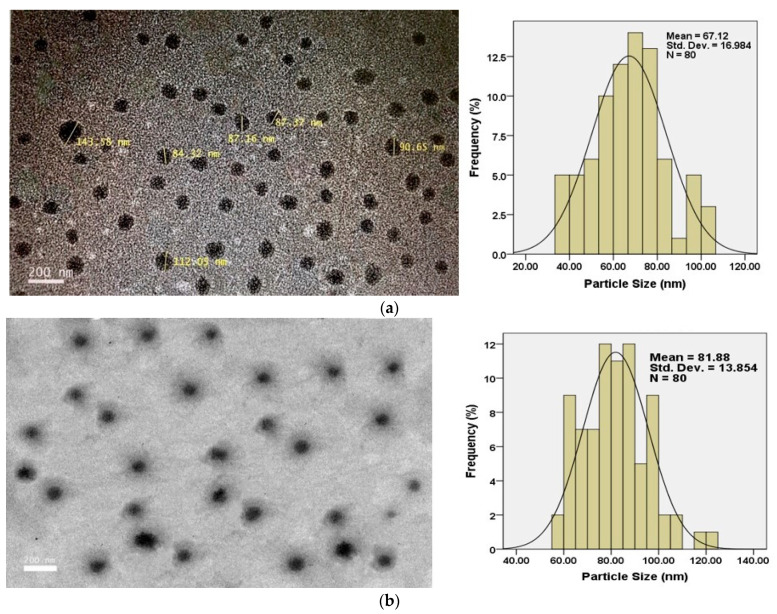
HRTEM micrographs of (**a**) Sorafenib-loaded chitosan nanoparticles (SF-CS) and (**b**) folate-conjugated, Sorafenib-loaded chitosan nanoparticles (SF-CS-FA).

The morphology of the nanoparticles was observed by the High-Resolution Transmission Electron Micrograph (HRTEM). Figure 8a shows that the SF-CS nanoparticles were found to be the spherical and uniformly dispersed with a size of approximately 40–80 nm. On the other hand, the folic acid-conjugated, SF-CS-FA nanoparticles were found to be a larger spherical shape of about 60–130 nm (Figure 8b). The mean size of SF-CS is 67.1 ± 16.9 nm compared to 81.9 ± 13.9 nm for SF-CS-FA. The latter exhibited a higher size due to the folic acid coating. Both nanoparticles were found to be uniformly distributed in the copper grid without any debris that can be clearly observed. The spherical shape and no debris were the indications of a good synthesis process of the nanoparticles. The morphology of TEM images also clearly shows the smooth surface of the synthesized nanoparticles, without any aggregation.

### 3.6. Fourier Transform Infrared Spectroscopy

**Figure 9:**
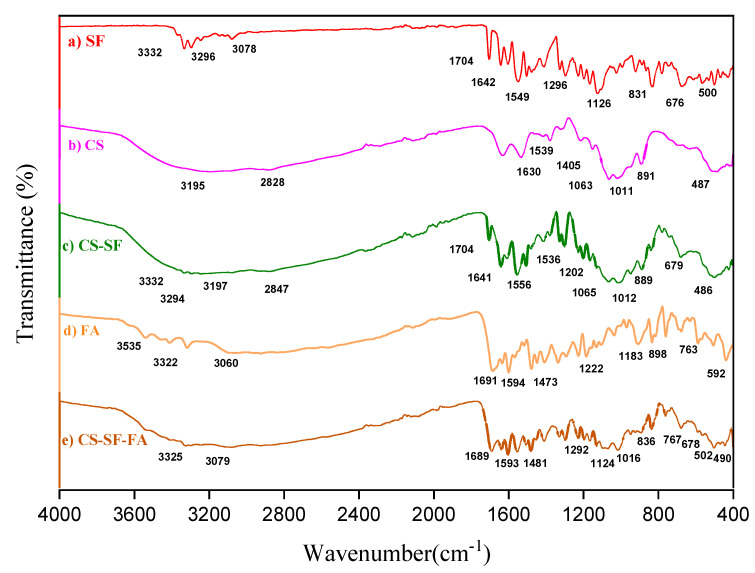
FTIR spectrum of SF (**a**), CS-NPs (**b**), SF-CS NPs (**c**), FA (**d**), and SF-CS-FA NPs (**e**).

Fourier transform infrared spectroscopy (FTIR) spectra of SF, CS-NPs, SF-CS, and SF-CS-FA are shown in Figure 9. The absorption bands were measured from 400 to 4000 cm^−1^.

The FTIR spectra of free Sorafenib (Figure 9a) shows two characteristic bands at 3332 and 3296 cm^−1^ due to the N-H stretching. Besides, a band at 3078 cm^−1^ is related to the C-H stretching band and at 1740 cm^−1^ is the characteristic band of the amide C=O group. A band at 1296 cm^−1^ and 1126 cm^−1^ is due to the presence of the C-O group attributed to the carboxylic acid. These bands were found in SF-CS NPs (Figure 9c) at 3332, 3294, 1704, 1641, 1556, and 1202 cm^−1^. A band at 679 cm^−1^ in SF-CS nanoparticles is attributed to C-Cl stretching of the SF drug. This observation is similar to that of previous works [41,42].

Figure 9b shows the FTIR spectrum of CS with several bands at 3195, 2828, 1630, 1539, 1063, and 1011 cm^−1^ and these bands are observed in Figure 9c when the SF drug was incorporated with CS. Figure 9c shows that the broadband at 3197 cm^−1^ corresponds to the stretching vibration of chitosan with combined bands of NH_2_ and OH groups. A band at 2847 cm^−1^ represented the C–H stretching vibration. The bands at 1536 and 1065 cm^−1^ are attributed to the CO-NH_2_ and NH_2_ groups of chitosan. Furthermore, a band at 1012 cm^−1^ represents the P=O stretching vibration of phosphate groups. Moreover, the FTIR bands of chitosan at 889 and 486 cm^−1^ are also observed in SF-CS NPs, representing the C=N stretching vibration in the chitosan.

When SF-CS-FA nanoparticles were formed (Figure 9e), a broad absorption band between 3550–3250 cm^−1^ and 3200–2500 cm^−1^ corresponding to the amine stretching in the C–H and O–H stretching in FA (Figure 9d), respectively, was observed. In Figure 9e, the band at 1292 cm^−1^ and 1124 cm^−1^ is due to the presence of the C-O group of carboxylic acid existing in SF and at 678 cm−^1^ attributed to C-Cl stretching of the SF drug. These bands indicate drug loading in the SF-CS-FA nanoparticles. The absorption band of C=O of the carboxylic acid of FA appears at 1689 cm^−1^. The C–O stretching vibration appears at 1481, and 836 cm^−1^, indicating the coating of FA on the nanoparticles. The formation of new bands at 1593 cm^−1^, and 767cm^−1^ corresponds to the aromatic C-C and aromatic C-H groups, respectively, indicating the presence of folic acid. Similar characteristics were also found by İnce et al., 2020 [43]. The band at 1016 cm^−1^ is due to the stretching vibration of the P=O and 490cm^−1^ was representing the C=N stretching of chitosan.

### 3.7. Thermogravimetric and Differential Thermogravimetric Analyses

**Figure 10:**
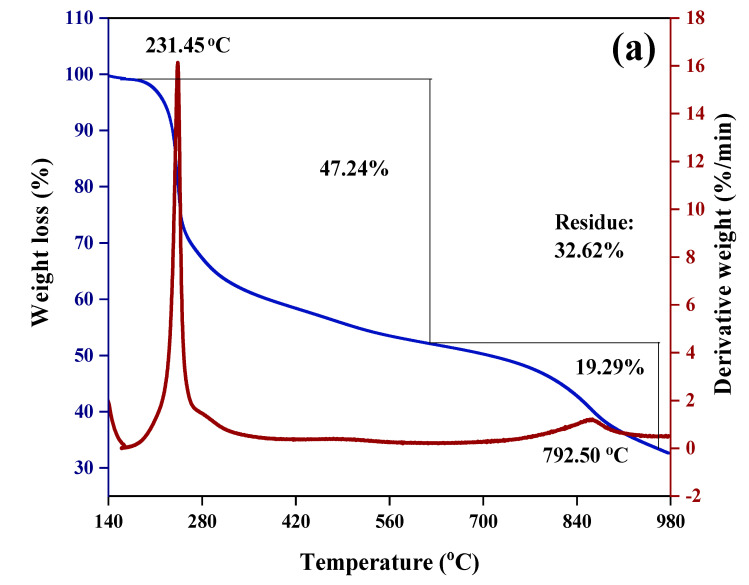

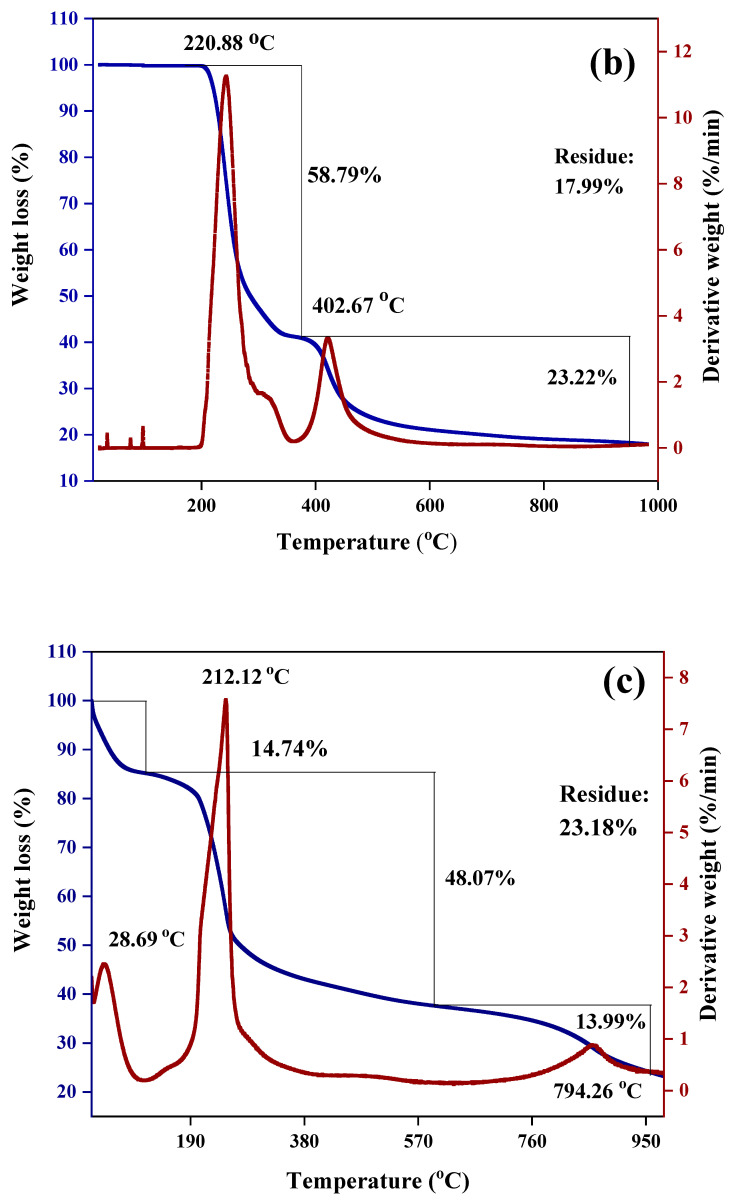

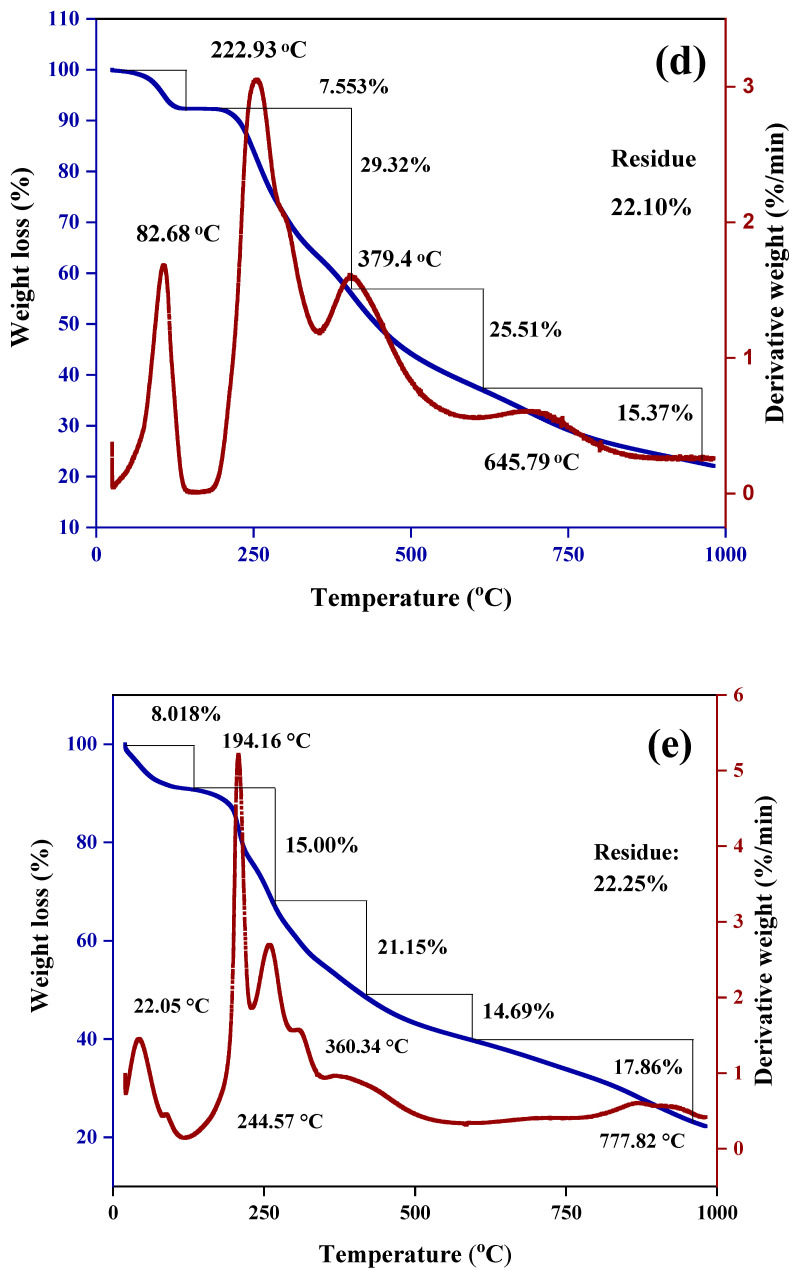
TGA/DTG thermograms of (**a**) CS-NPs, (**b**) SF, (**c**) SF-CS, (**d**) FA, and (**e**) SF-CS-FA.

The thermal properties of all the samples were investigated by thermogravimetric and differential thermo-gravimetric (TGA/DTG) thermal analyses. The TGA/DTG thermograms of the synthesized nanoparticles SF-CS, SF-CS-FA are shown in Figure 10. In Figure 10a, CS-NPs shows the weight loss in two stages at 231.5 and 792.5 °C with a percentage mass loss of 47.3 and 19.3%, respectively, due to the release of the water molecule and the decomposition of chitosan by hydrogen bond loss, respectively. Moreover, 58.8% weight loss was observed at 220.9 °C for the Sorafenib drug, indicating the decomposition of SF (Figure 10b). Furthermore, SF-CS nanoparticles show three stages of weight loss. In the first stage, the weight loss occurred at around 14.8% due to the release of the water molecule. The second stage of the weight loss of 48.1% at 212.2 °C is due to the decomposition of chitosan and the third stage at 794.3 °C with 13.9% of weight loss, is due to the decomposition of CS and SF, therefore showing the higher thermal stability of SF in the SF-CS nanoparticles compared to the SF drug alone (Figure 10c).

Figure 10d shows that at 82.68, 222.9, 379.5, and 645.8 °C, the weight loss of FA was found to be 7.6, 29.4, 25.6, and 15.4, respectively. For SF-CS-FA, the weight loss of 8.1, 15.0, 21.2, 14.7, and 17.9% was observed at 22.05, 194.2, 244.6, 360.4, and 777.9 °C, respectively. The weight loss at 194.2 °C represented the decomposition of SF, while the one at 244.6 and 360.4 °C is due to the decomposition of FA. At 777.9 °C, the weight loss is due to the decomposition of CS and SF_._ These results indicate the presence of a coating layer of FA in the nanoparticles with high thermal stability (Figure 10e). The residue for all the samples was found to be 17–32%, which indicates the higher stability of all the samples.

### 3.8. In-Vitro Drug Release

**Figure 11:**
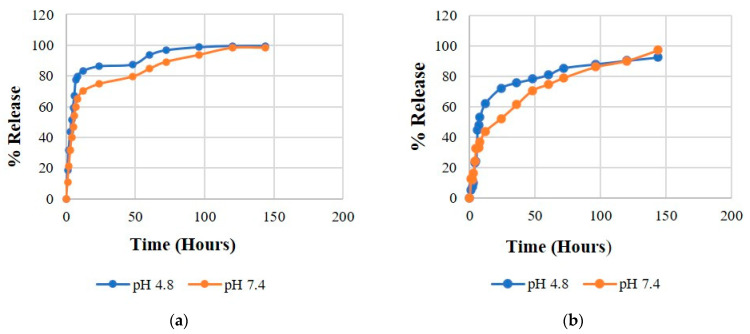
Release profiles of SF from its (**a**) SF-CS and (**b**) SF-CS-FA nanoparticles at pH 7.4 and 4.8 buffer solutions.

The delivery systems with sustained drug release are desirable to prolong the therapeutic effect of the drug after administration. The sustained release of SF will extend the duration of action, maintain constant drug level, minimize the systemic side-effects, and thus reduce the dosing frequency and increase efficacy. To determine the release of SF from SF-CS and SF-CS-FA NPs, in vitro release was simulated in phosphate-buffered saline (pH 7.4) and acetate buffered saline (pH 4.8) solutions. The two different pH conditions, 7.4 and 4.8, were used to mimic the physiological cell conditions and the acidic tumor environment, respectively. The release behavior of SF from the nanodrug delivery systems is shown in Figure 11, which depicts characteristics of the sustained release. A complete release was achieved after 100 h for both NPs systems. Based on Figure 11a,b, both SF-CS and SF-CS-FA nanoparticles showed the sustained release behavior of SF, which indicated the stability of SF within the nanoparticles. Figure 11a shows that at 72 h, 89.2% of SF released was achieved at pH 7.4 compared to 96.8% at pH 4.8 at the same time point for the SF-CS samples. After 120 h, the drug release was achieved at around 98.3% at pH 7.4 and 99.8% at pH 4.8. These release percentages indicate that SF is slightly more responsive to be released from its SF-CS at pH 4.8 than at pH 7.4.

Figure 11b shows the release of SF from its SF-CS-FA sample. About 78.9% of the drug was released in pH 7.4 solution compared to 85.4% in pH 4.8 at 72 h. At 120 h, 89.1% of the drug was released in pH 7.4 compared to 91.7% in pH 4.8. The SF release profile indicates that SF is more responsive to be released at pH 4.8 than pH 7.4 in SF-CS-FA nanoparticles, indicating that both SF-CS and SF-CS-FA nanoparticles are suitable for drug delivery purposes. Both NPs followed a slow-release initially, followed by a rapid release and ended with a steady release. The release profiles in Figure 11 indicate that in both NPs, the higher SF maximum percentages of release occurred in pH 4.8 solution than at pH 7.4 after 100 h. The release percentages of SF from its SF-CS-FA nanoparticles were slightly slower compared to its SF-CS nanoparticles. This might be due to the enhancement of bioactivity of the nanocarrier when it is conjugated with folic acid. FA might bind to the nanocarrier strongly, resulting in a slower breakdown in the buffer solution and slightly slower release of the SF.

### 3.9. Release Kinetics Study

**Figure 12:**
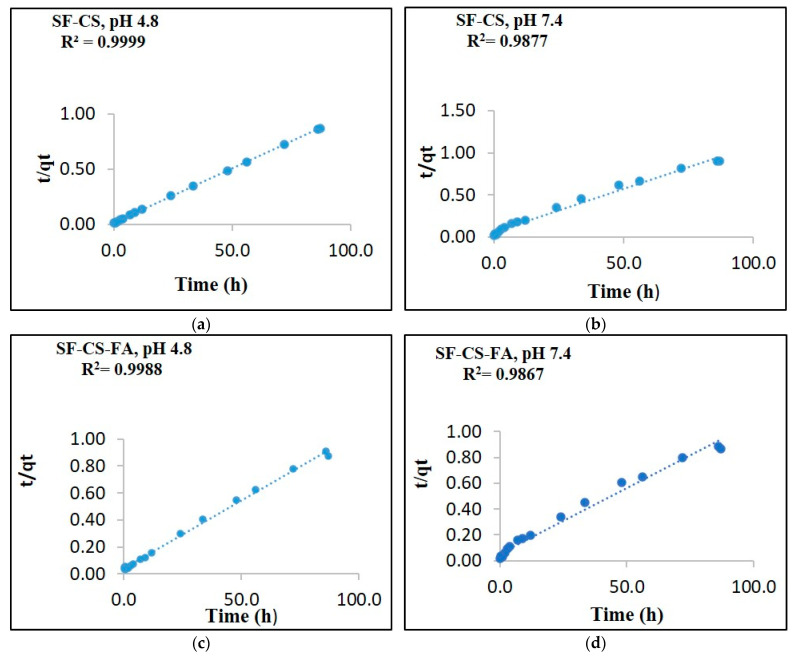
The data fitting of SF release from its SF-CS nanoparticles at pH 4.8 (**a**), pH 7.4 (**b**) and its SF-CS-FA nanoparticles at pH 4.8 (**c**), pH 7.4 (**d**) using the pseudo-second-order kinetics models.

The drug release from nanocarriers is controlled by various physicochemical factors, which require a mathematical release model to describe. To develop an effective nanodrug delivery system, it is important to determine its drug release profiles, to identify the process of drug release from the system. An ideal delivery system should follow the kinetics model to identify the nanocarrier’s drug release behavior. The release kinetics of SF drug from its nanoparticles were characterized using several kinetic models by ultraviolet-visible spectroscopy, using PBS solutions of pH 7.4 and 4.8. The SF drug release from the nanosystems was analyzed by curve fitting to various kinetic model-dependent methods, including the pseudo-first-order and pseudo-second-order kinetics as well as mathematical models including Higuchi, Hixson–Crowell and Korsmeyer–Peppas (Figure 12). A nanocarrier system that follows the pseudo-second-order kinetics refers to the process wherein the rate of release depends on the concentration of drug in the nanodelivery system. As a result, the drug is released under a constant and consistent flow.

Drug persistence in blood levels will also be constantly maintained throughout the delivery period. The six different kinetics models were applied to determine the kinetics of the drug release. Based on the fittings of the six models, the pseudo-second-order model represented a high correlation coefficient to the experimental data. Similar outcomes were obtained on both SF-CS and SF-CS-FA NPs in both pH 7.4 and 4.8 solutions. Therefore, the pseudo-second-order model has been considered a suitable model to describe the release kinetics of SF drug from both nanoparticles. In this experiment, the kinetics analysis was done in pH 7.4 and 4.8 solutions for both SF-CS and SF-CS-FA samples. In both pH 7.4 and 4.8 solutions, the SF release from the nanoparticles was found to be governed by the pseudo-second-order kinetics model, which represents a good delivery condition. The release of the drug from the nanocarrier systems pursued drug concentration-dependence, which means the rate of release will be controlled by SF concentration on both carrier systems [44].

The linear form of the pseudo-second-order kinetic equation can be represented as:

(3) $$t/q_{t}=\;1/K_{2}q_{e}^{2}+\;t/q_{e}$$

where q_e_ is release in equilibrium and q_t_ is the released drug at any time (t); K_2_ is the pseudo-second-order rate constant (mg/min).

Table 5 indicates the correlation coefficients (R^2^) obtained by fitting the Sorafenib release data from the SF-CS-NPs and SF-CS-FA NPs in PBS solutions at pH 7.4 and 4.8. Figure 12 shows no significant difference in the kinetic before and after coating with folic acid in both pH 4.8 and 7.4 PBS solutions. This indicates that SF’ release kinetics from both nanoparticles are similar. However, in pH 4.8 solution, both nanoparticles have slightly better release. The correlation coefficients at pH 4.8 indicated that the nanoparticles are more responsive at that pH, which is a good characteristic of nanodelivery for anti-cancer drugs.

### 3.10. In Vitro Cytotoxicity Studies

Cytotoxicity studies were conducted to determine the toxicity of the nanodelivery systems loaded with SF anticancer agents. The cytotoxicity studies were carried out by treating the normal HDFa dermal fibroblast cells, liver cancer-HepG2 cell lines, and colorectal cancer–HT29 cell lines with chitosan (CS), pristine sorafenib (SF), SF-CS nanoparticles, and SF-CS-FA nanoparticles. The samples were dosed at various concentrations 0.0, 1.25, 3.1, 6.25, 12.5, 25.0, 50.0, and 100.0 μg/mL individually to all the cell lines and incubated for 72 h. The half-maximal inhibitory concentrations (IC_50_) values of all the samples are shown in Table 6.

#### 3.10.1. In Vitro Cytotoxicity Study of Normal Human Dermal Fibroblast Adult Cells 

**Figure 13:**
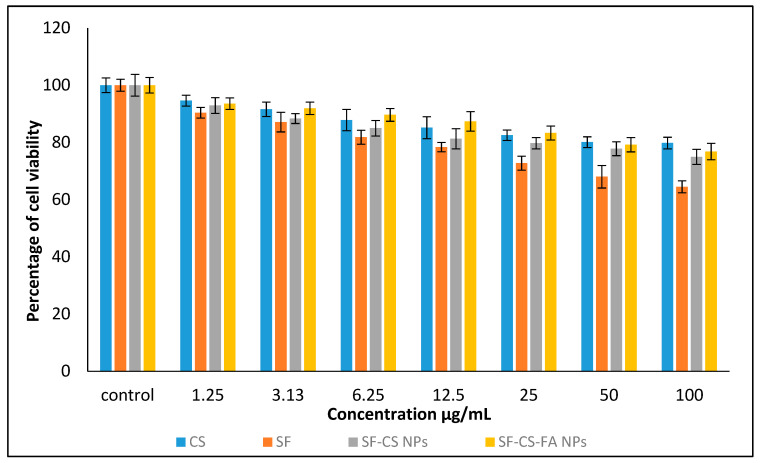
Cytotoxicity assay of chitosan, pristine sorafenib, CS-sorafenib, and CS-sorafenib-folic acid nanoparticles against normal HDFa dermal fibroblast cells at 72 h. Values are expressed as mean ± SD of triplicates. The significant differences were determined using the one-way ANOVA followed by Duncan’s Multiple Range Test.

Figure 13 shows the percentage of HDFa cells viability after a 72-h incubation period. The samples were found to be negligibly cytotoxic to HDFa dermal fibroblast cells. This suggests that the synthesized nanoparticles are biocompatible to the normal cells even at a high dose, which makes them suitable for treating the liver and colon cancer cells without affecting healthy cells. Statistical analysis was conducted using the ANOVA and Duncan’s Multiple Range Test. A notable difference in cell death was found in SF drug alone compared to all other NPs at the individual level from 1.25–100 µg concentrations, indicating that the NPs are safer to use compared to the drug alone in treating cancer cells.

#### 3.10.2. Anticancer Activity against Liver Cancer, HepG2 Cells 

**Figure 14:**
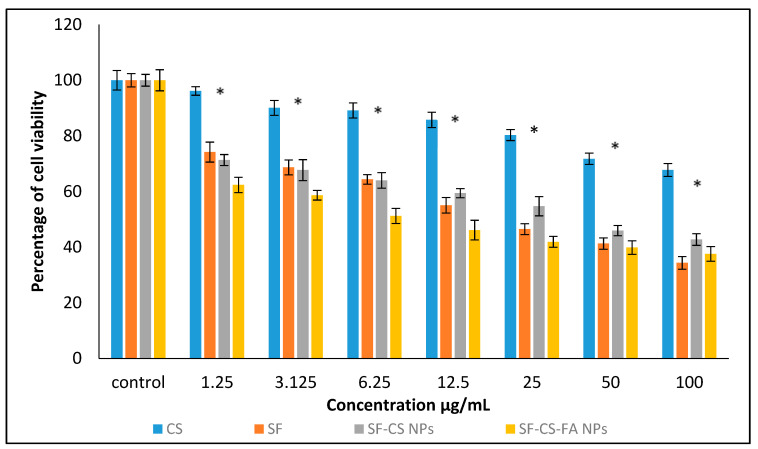
Cytotoxicity assay of CS, SF, SF-CS, and SF-CS-FA nanoparticles against HepG2 cells at 72 h of incubation. Values are expressed as mean ± SD of triplicates. The significant differences (*p* < 0.05) * were determined using the one-way ANOVA followed by Duncan’s Multiple Range Test.

The anticancer action of chitosan (CS), pristine sorafenib (SF), SF-CS, and SF-CS-FA nanoparticles was tested on liver cancer–HepG2 cell lines. Figure 14 shows the results of different concentrations of all individual samples incubated with HepG2 cells for 72 h. The CS alone, at concentrations of 1.25–100 µg, shows negligibly inhibitory activity against the HepG2 cell line. Although CS only showed toxicity at higher concentrations in the fibroblast cells and the cancer cell, in this experiment, based on the IC_50_ value for chitosan, it was found that it is non-toxic to the fibroblast and HepG2 cancer cells. The non-susceptibility of the normal and cancer cells towards CS indicates that it is a good carrier material. The IC_50_ of SF against the HepG2 cells was found to be around 21.6 ± 1.0 μg/mL, while the IC_50_ of the SF-CS and SF-CS-FA nanoparticles was found to be 20.3 ± 1.5 μg/mL and 14.5 ± 2.5 μg/mL, respectively. The amount of Sorafenib present in IC_50_ of the nanoparticles was calculated from the percentage of SF drug loading, which is 18.2 ± 1.4% for SF-CS and 19.9 ± 1.3% for SF-CS-FA nanoparticles. The lower IC_50_ values of both NPs indicates that the nanoparticles of SF-CS at 20.3 ± 1.5 μg/mL and SF-CS-FA at 14.5 ± 2.5 μg/mL have better anticancer action and cytotoxicity effect compared to the pristine free Sorafenib drug.

#### 3.10.3. Anticancer Activity against Colorectal Cancer, HT29 Cells

**Figure 15:**
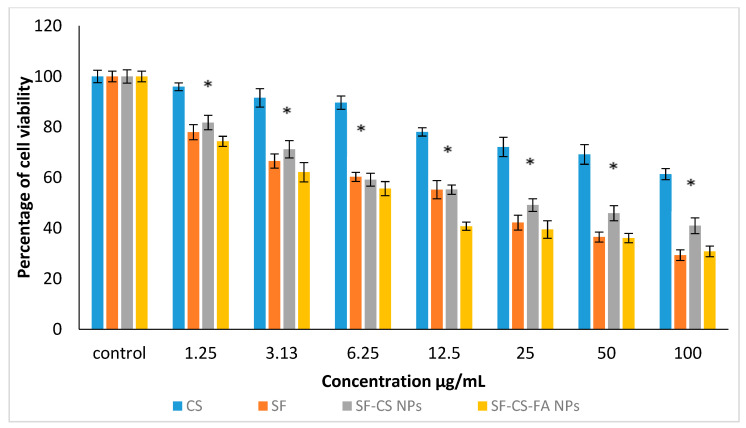
Cytotoxicity assay of CS, SF, SF-CS, and SF-CS-FA nanoparticles against HT29 cells at 72 h of incubation. Values are expressed as mean ± SD of triplicates. The significant differences (*p* < 0.05) * were determined among untreated HT29 using the one-way ANOVA followed by Duncan’s Multiple Range Test.

The anticancer action of CS, SF, SF-CS, and SF-CS-FA NPs was also observed on colorectal cancer, HT29 cells, shown in Figure 15. Several concentrations of all the samples were incubated with HT29 cells for around 72 h. The IC_50_ value of the pristine Sorafenib was found to be 16.8 ± 1.8 μg/mL against the HT29 cells. The chitosan nanocarrier alone showed negligible toxicity in the colorectal cancer cell for the concentrations of 1.25–100 µg. In this experiment, the IC_50_ value for chitosan was found to be non-toxic to the fibroblast and HT29 cancer cells. The IC_50_ of the SF-CS and SF-CS-FA nanoparticles were found to be 15.9 ± 2.0 μg/mL and 13.0 ± 1.3 μg/mL, respectively. The efficacious IC_50_ of Sorafenib present in the nanoparticles was calculated from the percentages of SF loading, which is 18.2 ± 1.4% and 19.9 ± 1.3% for SF-CS and SF-CS-FA, respectively. These results demonstrated that the lower IC_50_ of SF-CS at 15.9 ± 2.0 μg/mL and SF-CS-FA at 13.0 ± 1.3 μg/mL had better anticancer activity compared to the relatively higher IC_50_ value in its SF counterpart. This also reveals that both SF-CS and SF-CS-FA nanoparticles have a better cytotoxicity effect toward HT29 cells compared to SF alone.

SF-CS and SF-CS-FA nanoparticles showed anticancer effect towards HepG2 and HT29 cell lines in a dose-dependent manner, which indicates that increase in drug concentration will increase anticancer activity. The IC_50_ values indicates that the synthesized SF-CS and SF-CS-FA nanoparticles have a better anticancer effect compared to the counterpart, free forms of SF.

Due to better cytotoxicity of the HepG2 and HT29 cell lines compared to the normal fibroblast cells, as shown in this work, Sorafenib-loaded SF-CS and SF-CS-FA nanoparticles could be effective anticancer drug delivery systems in the future. However, further cellular studies need to be conducted to further explore the efficiency of these NPs at the cellular level, such as cellular uptake, flow cytometric analysis, and in-vivo cytotoxicity studies.

## 4. Conclusions

Nanocarrier drug delivery systems for therapeutic applications have currently attracted significant attention in various cancer treatments. Chemotherapy is a popular option to treat liver and colon cancer—the drug spreads throughout the body, including healthy cells, causing the death of normal cells. Nanocarriers-based drug delivery systems such as nanoparticles has some benefits over conventional drug delivery systems. This is because the host of the nanodrug delivery system can protect the drug from premature degradation, and prevent it from premature interaction with another non-targeted biological environment, such as protein, lipids, and other biological components. Moreover, they can enhance drug absorption in targeted areas, especially tumors. In a nutshell, nanocarriers can be the future of nanodrug delivery systems for human liver and colon cancer treatment. This experiment emphasized the development of Sorafenib-loaded chitosan nanoparticles and their folate-conjugate nanoparticle formulation, which could potentially be used as an anticancer drugs vector. The properties of good particle size, shape, sustained release, and enhanced anticancer activities of the drugs make these nanodelivery systems more effective compared to the drug alone. In this work, Sorafenib was entrapped into chitosan nanoparticles. Obtaining a proper drug-to-nanocarrier ratio was a challenge, which was finally accomplished after parametric optimization. These delivery systems were successfully developed for the delivery of Sorafenib in HepG2 liver cancer and HT29 colon cancer cells. Both nanoparticles show a near-spherical shape and exhibit relatively slower drug release at pH 4.8, compared to pH 7.4 PBS buffer solutions. Both SF-CS and SF-CS-FA NPs have shown remarkable anticancer effects compared to its free SF in the cell lines of HepG2 and HT29. Both nanodrug delivery systems showed highly promising behaviors for liver and colon cancer due to their low cytotoxicity, high drug loading capacity, and efficient anticancer ability. The MTT assay studies have shown that both nanoparticles displayed a higher therapeutic efficiency towards HepG2 cells and HT29 cells. Therefore, SF-CS and SF-CS-FA nanoparticles opened up a promising delivery vehicle for liver and colon cancers. However, further investigation of the potential of NPs such as major cellular studies, cellular uptake, and in vivo studies should be conducted to study the efficiency of the synthesized NPs.

## 5. Future Aspects

Future aspects of the study will include LDH assays, Caspase activity assays, cellular uptake, western blots, flow cytometric analysis, and in vivo studies for both SF-CS and SF-CS-FA nanoparticles. However, these studies are not in the scope of this manuscript.

## Figures and Tables

**Table 1:** The effect of size, poly dispersity index, encapsulation efficiency (%) and loading content (%), and zeta potential on chitosan: Sorafenib ratios. Optimal parameters are shown in bold.

Sample	Chitosan: Sorafenib ratio (mg/mL)	Size (nm)	PDI	EE (%)	LC (%)	Zeta Potentials (mV)
SF-CS NPs	5:0	10.5 ± 9.6	0.22	0.0 ± 0.0	0.0 ± 0.0	7.9 ± 0.9
	5:1	27.2 ± 12.6	0.19	20.2 ± 1.2	7.7 ± 0.5	8.7 ± 0.4
	5:2	30.8 ± 11.9	0.23	69.8 ± 3.3	12.2 ± 0.2	19.8 ± 0.4
	**5:3**	**76.3 ± 13.7**	**0.28**	**83.7 ± 2.4**	**18.2± 1.3**	**31.5 ± 0.6**
	5:4	67.9 ± 11.2	0.10	79.9 ± 1.9	14.8 ± 1.0	32.8 ± 0.5
	5:5	28.7 ± 7.8	0.45	49.8 ± 0.9	7.6 ± 0.6	37.7 ± 0.4
	5:6	15.4 ± 9.8	0.68	19.4 ± 1.3	6.9 ± 0.1	37.6 ± 0.1
	5:7	27.8 ± 5.0	0.92	3.7 ± 1.1	12.9 ± 0.4	31.8 ± 0.3
	5:8	14.9 ± 5.9	2.07	8.8 ± 0.5	6.0 ± 0.6	25.4 ± 0.7
	1:3	18.5 ± 7.3	0.09	9.8 ± 0.6	1.9 ± 0.0	17.6 ± 0.9
	2:3	47.6 ± 3.9	0.05	6.9 ± 0.9	8.9 ± 0.5	11.3 ± 0.8
	4:3	52.5 ± 4.2	1.19	9.0 ± 1.1	3.2 ± 0.6	27.9 ± 0.5
	6:3	66.3 ± 8.2	0.34	55.9 ± 2.6	12.9 ± 1.7	21.2 ± 0.5
	7:3	139.2 ± 2.7	0.21	34.9 ± 2.3	14.4 ± 1.7	34.4 ± 0.7
	8:3	120.2 ± 2.9	0.49	45.6 ± 1.9	9.9 ± 1.2	29.1 ± 0.4
	9:3	201.0 ± 3.8	5.07	19.7 ± 1.2	6.9 ± 1.8	24.7 ± 0.5

**Table 2:** The effect of size, poly dispersity index, encapsulation efficiency (%) and loading content (%), and zeta potential of the SF-CS nanoparticles after coating them with folic acid for the formation of SF-CS-FA nanoparticles. Optimal parameters are shown in bold.

Sample	CS: SF (mg/mL)	Folic Acid (g/L)	Size (nm)	PDI	EE%	LC%	Zeta Potentials (mV)
SF-CS-FA	**5:3**	0.1	45.8 ± 4.2	0.05	2.00 ± 0.0	0.0 ± 0.0	27.6 ± 1.6
		0.2	49.7 ± 7.2	0.09	7.9 ± 1.0	0.7 ± 0.5	21.1 ± 1.2
		0.3	54.5 ± 9.3	0.11	12.8 ± 1.3	9.2 ± 0.2	27.2 ± 2.1
		0.4	57.8 ± 10.2	0.16	13.7 ± 1.4	8.0 ±0.3	25.7 ± 1.3
		0.5	62.6 ± 12.9	0.13	15.8 ± 1.4	8.8 ± 1.7	37.4 ± 2.1
		0.6	61.5 ± 13.7	0.21	19.9 ± 1.9	7.56 ± 0.6	39.8 ± 0.7
		0.7	78.7 ± 11.1	0.20	49.9 ± 1.6	11.13 ± 1.1	38.5 ± 0.6
		**0.8**	**81.7 ± 12.9**	**0.24**	**87.9 ± 1.1**	**19.9 ± 1.4**	**37.7 ± 1.4**
		**0.9**	**89.9 ± 10.6**	**0.26**	**88.9 ± 1.5**	**16.7 ± 1.6**	**36.4 ± 1.5**
		**1.0**	**91.5 ± 9.9**	**0.31**	**89.8 ± 1.6**	**17.9 ± 1.0**	**37.2 ± 0.3**
		1.2	140.6 ± 9.7	0.50	76.2 ± 1.9	15.2 ± 1.5	27.4 ± 0.5
		1.4	165.8 ± 5.5	0.90	68.6 ± 1.1	11.8 ± 1.1	22.6 ± 1.7
		1.6	180.5 ± 10.1	1.89	55.9 ± 1.2	9.7 ± 0.7	23.8 ± 1.5
		1.8	289.9 ± 7.8	2.67	64.9 ± 0.3	7.7 ± 1.7	26.7 ± 0.5
		2.0	298.1 ± 5.9	3.83	75.6 ± 1.9	4.9 ± 1.2	29.7 ± 0.5

**Table 3:** The percentages of LC and EE of SF-CS and SF-CS-FA NPs at the optimum amount of CS, SF, and FA.

Synthesized Nanoparticle	Loading-Content (%)	Encapsulation-Efficiency (%)
SF-CS	18.2 ± 1.3	83.7 ± 2.4
SF-CS-FA	19.9 ± 1.4	87.9 ± 1.1

**Table 4:** Elemental compositions; atomic% and weight% of the nanoparticles obtained by the EDX analysis.

Atomic and Weight %	Element (%)	SF-CS NPs	SF-CS-FA NPs	CS-NPs
**Atomic%**	C	46.9	48.9	36.4
	N	7.6	14.2	-
	O	38.6	32.5	50.1
	F	4.2	2.9	-
	P	2.4	0.5	7.6
	Cl	0.6	0.3	-
	S	-	0.5	-
	Na	-	-	5.8
**Weight%**	C	39.1	42.4	27.2
	N	6.4	14.7	2.6
	O	40.1	35.3	39.2
	F	5.6	3.9	-
	P	5.3	1.2	14.7
	Cl	1.6	0.5	-
	S	0.8	1.3	-
	Na	1.3	0.6	13.96

**Table 5:** Correlation coefficient value (R^2^) of kinetics release of SF from its SF-CS and SF-CS-FA nanoparticles into PBS solutions at pH 7.4 and 4.8 using the first-order, pseudo-first-order, pseudo-second-order kinetics, Higuchi, Hixon-Cromwell, and Korsmeyer Peppas models and the pseudo-second-order rate constant, K_2_ (mg/min).

Sample	Max Release (%)	Pseudo-First-Order	Pseudo-Second-Order	First-order	Higuchi	Korsmeyer-Peppas	Hixon-Crowell	Pseudo Second Order Rate Constant (mg/min) K_2_
SF-CS	98	0.0388	0.9977	0.3971	0.8246	0.8677	0.4885	7.2 × 10^−3^
SF-CS-FA	88	0.8053	0.9877	0.3970	0.8246	0.8677	0.4533	8.5 × 10^−3^
SF-CS	99	0.8053	0.9999	0.3961	0.8246	0.8677	0.4533	7.6 × 10^−6^
SF-CS-FA	93	0.8053	0.9988	0.3970	0.8246	0.8677	0.4533	7.2 × 10^−6^

**Table 6:** The half-maximal inhibitory concentration (IC_50_) value for chitosan (CS), pristine sorafenib (SF), SF-CS, and SF-CS-FA nanoparticles tested on normal HDFa cells, HepG2 and HT29 cell lines.

Nanoparticles	HDFa	HepG2	HT29
IC_50_ (μg/mL)			
Chitosan	N.C	N.C	N.C
Sorafenib	N.C	21.6 ± 1.0	16.8 ± 1.8
SF-CS	N.C	20.3 ± 1.5	15.9 ± 2.0
SF-CS-FA	N.C	14.5 ± 2.5	13.0 ± 1.3

Abbreviation: N.C. = No cytotoxicity.

## Abbreviations

NPs	Nanoparticles
CS-NPs	Chitosan nanoparticles
CS	Chitosan
DLS	Dynamic light scattering
HCL	Hydrochloride
TEM	Transmission electron microscopy
TPP	Sodium Tripolyphosphate
UV-Vis	Ultraviolet-visible spectroscopy
DMSO	dimethyl sulfoxide
MTT	methyl thiazol tetrazolium bromide
NaOH	sodium hydroxide
nm	nanometer
PBS	phosphate-buffered saline
TPP	(sodium) tripolyphosphate
EPR	Enhanced Permeability and Retention
EDX	Energy Dispersive X-ray
FESEM	Field Emission Scanning Electron Microscopy
FTIR	Fourier Transform Infrared
XRD	X-ray spectroscopy
EE	Encapsulation Efficiency
LC	Loading Content
M	molar
mg	milligram
min	minute(s)
ml	milliliter
Hep G2	—Hepatocellular Carcinoma cell
HT29	Colorectal Adenocarcinoma Cell Lines
